# Names and Theories

**Published:** 1905-02-25

**Authors:** 


### NAMES AND THEORIES.

In his recent address as President of the British Balneological and Climatological Society Mr. Bowen Davies gave an appropriate illustration of the evils which may arise from applying to diseased conditions names which carry a theoretical significance. Thus, certain chronic and more or less deforming changes in the joints are commonly named rheumatoid arthritis and often rheumatic gout, and as a result the victims of these processes are frequently subjected to dietetic and medicinal treatment based upon the etiological views which these names suggest. Against such measures Mr. Davies, upon the basis of a long experience, enters an emphatic protest. He points out, what is indeed admitted by all well-informed physicians, that treatment on these lines so far from possessing remedial virtue is decidedly detrimental. Yet it cannot be doubted that the persons really responsible for these prejudicial therapeutic proceedings are the inventors and advertisers of the names in question. The names, whatever qualifications may be attached to them by their original sponsors, carry suggestions which inevitably prompt lines of treatment now known to be ill-directed, and the evil is apt to be continued through succeeding generations. The selection of appropriate names for the various manifestations of disease is as difficult and delicate a process as the choice of names in other departments of observation. But there is one commanding principle which demands universal recognition, and this is that the name shall not include theoretical considerations. If a name can be devised which is a succinct description of the prominent facts so much the better, but even an absolutely arbitrary term, such as the name of the physician or patient, is infinitely better than one which is based upon speculative or theoretical views. Time only too often upsets the theory even when the facts remain securely grounded. And over ill-devised names much time and temper have been spent in fruitless controversy, and on them much unwise practice has been framed.

